# DAG-informed regression modelling, agent-based modelling and microsimulation modelling: a critical comparison of methods for causal inference

**DOI:** 10.1093/ije/dyy260

**Published:** 2018-12-05

**Authors:** Kellyn F Arnold, Wendy J Harrison, Alison J Heppenstall, Mark S Gilthorpe

**Affiliations:** 1 Leeds Institute for Data Analytics, University of Leeds, Leeds, UK; 2 School of Medicine, University of Leeds, Leeds, UK; 3 School of Geography, University of Leeds, Leeds, UK

**Keywords:** causal inference, counterfactuals, directed acyclic graphs, agent-based modelling, microsimulation modelling

## Abstract

The current paradigm for causal inference in epidemiology relies primarily on the evaluation of counterfactual contrasts via statistical regression models informed by graphical causal models (often in the form of directed acyclic graphs, or DAGs) and their underlying mathematical theory. However, there have been growing calls for supplementary methods, and one such method that has been proposed is agent-based modelling due to its potential for simulating counterfactuals. However, within the epidemiological literature, there currently exists a general lack of clarity regarding what exactly agent-based modelling is (and is not) and, importantly, how it differs from microsimulation modelling—perhaps its closest methodological comparator. We clarify this distinction by briefly reviewing the history of each method, which provides a context for their similarities and differences, and casts light on the types of research questions that they have evolved (and thus are well suited) to answering; we do the same for DAG-informed regression methods. The distinct historical evolutions of DAG-informed regression modelling, microsimulation modelling and agent-based modelling have given rise to distinct features of the methods themselves, and provide a foundation for critical comparison. Not only are the three methods well suited to addressing different types of causal questions, but, in doing so, they place differing levels of emphasis on fixed and random effects, and also tend to operate on different timescales and in different timeframes.

**Key Messages**

- Microsimulation modelling and agent-based modelling are closely linked methodologically though historically distinct. The key features of agent-based modelling are agency and agent-to-agent interaction, which produce highly complex and ‘emergent’ properties.
- Directed acyclic graph (DAG)-based regression modelling directs greatest focus towards modelling mean structures (i.e. ‘fixed’ effects), whereas simulation approaches embrace complexity by focusing more on ‘random’ structures.
- Microsimulation modelling provides a bridge between DAG-informed regression modelling and agent-based modelling, which may be exploited to bring the mathematical robustness of graphical model theory to bear on simulation approaches.
- Agent-based modelling can provide a complimentary extension to DAG-informed regression methods in order to deal with scenarios involving greater complexity (e.g. in which the assumption of no ‘interference’ or spillover effects may be untenable).

## Introduction

Epidemiology, which entails the study of both the distribution and determinants of health and disease, is often considered the core science of public health.^1^ Whilst easy to conceptualize, it nevertheless remains difficult to practise. Population-level health patterns emerge from a complex, dynamic, multi-layered system, in which a multitude of different interrelationships operate^2^; this system is commonly referred to in the literature as a ‘complex system’, which is characterized by individual heterogeneity and autonomy, interdependence, spillover effects, adaptivity and evolution, feedback and threshold effects.^3^ Individuals move through space and time, interacting with and being influenced by other individuals, groups, social, economic and political constraints, and geography—to name but a few. Understanding the impact of individual behaviour and decision-making on population health trends—so that we are ultimately able to intervene to alter them beneficially—necessarily requires a *causal* understanding of those patterns and processes that are important, and at which spatial and temporal scales they operate.

The inherent complexity of such a system poses challenges to anyone attempting to model it; identifying and estimating causal effects creates additional challenges. Causation is a concept of which most, if not all, human beings have an intuitive understanding. Nevertheless, it is a complex phenomenon and remains largely inarticulable; despite thousands of years of philosophical discourse, there exists very little consensus as to what it is, how it can be defined^4^ and—perhaps most importantly for researchers—how it can be inferred within practical research applications.^5–10^ To address this, many methods have emerged across a range of different disciplines, though the current paradigm for causal inference in epidemiology relies primarily on the evaluation of counterfactual contrasts via statistical regression models informed by graphical causal models (often in the form of directed acyclic graphs, or DAGs) and their underlying mathematical theory.^5^ However, there have been growing calls for a more pluralistic approach to causal inference in the field,^5^^,^^6^^,^^11^ premised on the argument that there are numerous causal scenarios that do not lend themselves to representation by DAGs and subsequent statistical analyses.

Many authors have proposed more widespread adoption of ‘systems approaches’^2^^,^^3^^,^^12–15^—a somewhat nebulous term for a group of methods that may be used to study the nature of systems. In particular, several authors have identified agent-based models (ABMs) as promising tools for causal inference in complex systems, as they provide a framework for the simulation of counterfactuals.^15–17^ Perhaps due to agent-based modelling having primarily evolved within and been adopted by the ‘softer’ social sciences (e.g. sociology, political science), it remains relatively unfamiliar to epidemiological researchers; moreover, there appears to be little clarity regarding what exactly an ABM is (and is not) and, importantly, how it differs from other simulation models. For example, the recent work of Murray *et al*.^18^ demonstrated equivalence between the parametric g-formula (a statistical method based upon graphical model theory) and what the authors referred to as agent-based modelling, though, in actuality, it is more akin to microsimulation modelling. Whilst the distinction between ABMs and microsimulation models (MSMs) may seem self-evident to those who regularly use these methods and trivial to those who do not, it does in fact have important implications for how and under which circumstances each may be used, and thus is worth clarifying.

To this end, we seek to elucidate the distinction between microsimulation modelling and agent-based modelling for statistically minded researchers who may be relatively unfamiliar with them; moreover, we describe DAG-informed regression modelling for simulation-minded researchers. Because these methods have largely been confined to separate research disciplines, there exists little overlap in the knowledge about them and skills necessary for implementing them, despite calls for greater integration^2^^,^^13^^,^^15^^,^^17^; our paper aims to fill this gap. We begin by briefly explaining each method and its history, and go on to discuss how their separate evolutions have shaped the types of causal questions to which they are well suited to evaluating. We outline the primary philosophical and methodological similarities and differences between them, and conclude with a discussion regarding the implications of these similarities and differences for future causal analyses and opportunities for future methodological work.

## A brief history of methods

Historical context is key to understanding both the utility and the defining features of a particular method; therefore, we briefly recap the history of each method, with specific attention given to how it evaluates counterfactuals (see Box 1 for an explanation of counterfactuals).

**Box 1. A brief explanation of counterfactuals**

Counterfactuals

The counterfactual framework states that an event A may be considered a cause of an event Y if, contrary to fact, had A not occurred then Y would not have occurred.^4^ As an example, imagine that an individual, Alison, is driving to work and comes to a fork in the road. She chooses to go left, and arrives late for work. Upset, Alison declares ‘I should have gone right instead!’ What her statement implies is that her decision to go left at the fork in the road caused her to be late for work because had she gone right she would not have arrived late. Of course, there is no way to prove such a statement, as it would require Alison to simultaneously go both left and right and observe the outcome under each condition (to guarantee that the effect is not attributable to any other factor that differed between drives); nevertheless, the scenario demonstrates the utility of examining causal effects as counterfactual contrasts between two exchangeable units of analysis—those that are equivalent in every way except for the putative causal factor of interest.

### Graphical causal models and the formalization of counterfactuals

Causal models trace their roots back to 1918, with Sewall Wright’s invention of path analysis.^19^^,^^20^ They also have origins in structural equation models (SEMs), which emerged primarily in the social sciences (e.g. psychology) and represent groups of causally related variables (both observed and latent) as systems of simultaneous (linear) equations.^21^ However, both were subsumed at the turn of the century under the framework of non-parametric causal models by Judea Pearl in his seminal book *Causality*.^22^ These models are typically represented graphically as a set of nodes (variables) connected by a set of edges (representing statistical dependencies), although neither the magnitude nor functional form of these dependencies are implied or constrained.^23^ A special subset of such graphs—DAGs—are perhaps the most well known.

**Figure 1.**
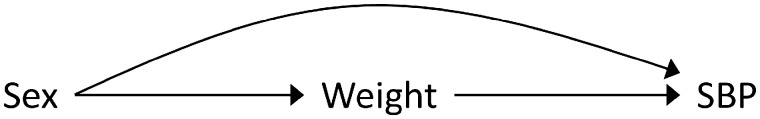
Directed acyclic graph (DAG) depicting the hypothesized causal relationships between sex, weight and systolic blood pressure (SBP).

A DAG is a graphical causal model in which all edges are unidirectional (hence ‘directed’); these directed edges represent direct causal effects. A path is a sequence of edges connecting two nodes, and there may be multiple paths connecting any two nodes. A causal path is one in which all directed edges flow in the same direction, indicating that the statistical dependency that exists between the nodes is causal in nature. Importantly, no causal paths may exist from any node back to itself (hence ‘acyclic’).^23^^,^^24^ A node may be either endogenous (having at least one direct cause represented in the DAG) or exogenous (having none), and a DAG may be considered a ‘causal DAG’ if all common causes between any two nodes are represented in the graph.^24^ A simplified example is given in Figure 1, showing the hypothesized causal relationships between sex, weight and systolic blood pressure (SBP).

DAGs represent a given system as a number of variables connected by a series of causal pathways; combined with parametric assumptions, they may be thought of as representing the presumed data-generating process, i.e. the process by which any endogenous variable in the system obtains its value. Given the values for all exogenous variables, the value of any endogenous variable can be known. For example, if we knew the value of sex in Figure 1 (and assumed it was a causal DAG for ease of illustration, though in reality this is unlikely), we could also know the values of weight and SBP, because weight depends only upon sex for its value, and SBP in turn depends upon weight and sex.

Whilst identification of individual-level causal effects is generally agreed to be impossible in the real world within a counterfactual framework (i.e. ‘the fundamental problem of causal inference’), identification of *average* causal effects is possible and, indeed, forms the basis of a great deal of causal inference.^24^ Randomized–controlled trials (RCTs)—often considered the ‘gold standard’ for demonstrating causality—create exchangeable units of analysis by randomly assigning individuals to receive either the putative causal factor of interest or a standard alternative that acts as the reference (e.g. placebo control). Thus, although *individuals* within the study likely differ with respect to both measured and unmeasured characteristics that may affect the outcome of interest, randomization ensures that the distribution of such factors is broadly equivalent between the groups so that, on average, the two *groups* are exchangeable and thus any difference in average outcomes may be attributed to the hypothesized causal factor.^24^

DAGs are an incredibly powerful tool for statistical analyses because they provide the foundation for estimating counterfactual quantities from observed data; they have thus found a natural home amongst disciplines in which data collection and statistical analysis are considered paramount (e.g. epidemiology). Creating exchangeable units of analysis is trivial in a well-conducted RCT but more difficult to achieve with non-experimental data in which the putative causal factor of interest is not randomly assigned; simply comparing the average outcomes between those who were or were not exposed to that factor would, in general, not be sufficient for identifying an average causal effect, since the differences in outcomes might be attributable to other differences between the groups. However, in principle, identification of a causal effect could be achieved by comparing the outcomes amongst *subgroups* for which the distributions of relevant factors are broadly equivalent. Such subgroups would therefore be referred to as *conditionally* exchangeable (or exchangeable conditional on these factors).

The power of graphical model theory is that it provides a way of determining which variables are sufficient for guaranteeing conditional exchangeability for a given DAG, thereby formalizing counterfactual logic and facilitating what has been referred to as the ‘algorithmisation of counterfactuals’.^25^ Briefly, a set of variables is sufficient for guaranteeing conditional exchangeability if conditioning on that set blocks all ‘backdoor paths’ (i.e. spurious paths that induce statistical dependence due to one or more common causes—referred to as ‘confounding’) between the putative causal factor and outcome of interest whilst leaving all causal paths intact.^23^ In practice, this generally involves creating a regression model for the outcome that includes as covariates both the putative causal factor and a set of variables sufficient for removing bias due to confounding.^23^ In Figure 1, for instance, sex confounds the relationship between weight and SBP; therefore, if we wished to estimate the total causal effect of weight on SBP, we could estimate the parameters of the following regression model:

$$SBP=\beta_{0}+\beta_{1}\cdot weight+\;\beta_{2}\cdot sex+\varepsilon .$$

Assuming that the model has been correctly parameterized, we are able to interpret ${\hat{\beta }}_{1}$ as the estimated total causal effect of weight on SBP. In other words, for individuals of the same sex (i.e. conditionally exchangeable individuals), every unit-difference in weight corresponds to an expected difference in SBP of ${\hat{\beta }}_{1}$, on average. DAGs therefore provide a framework for using traditional statistical methods to estimate counterfactual quantities and average causal effects via the creation of conditionally exchangeable units of analysis.

### Microsimulation models, agent-based models and the simulation of counterfactuals

Microsimulation and agent-based modelling are closely linked methodologically and conceptually though historically distinct, which perhaps obfuscates where they in fact overlap and where they diverge. Both have roots in cellular automata,^26^ which first emerged in the 1940s and involve simulating the evolution of a collection of coloured cells within a grid at discrete time steps in accordance with a set of rules based on the states of neighbouring cells. From this, MSMs and ABMs evolved separately (primarily in economics and sociology, respectively) as more complex simulation methods; their development and implementation were greatly enabled by the advent of programmable electronic computers. Whereas both methods have been in use for approximately the last half-century—with Orcutt^27^ frequently credited as one of the founding fathers of the field of microsimulation and Schelling^28^ for agent-based modelling—the vast increases in computing power realized in the age of technology have rendered early implementations virtually unrecognizable in comparison to their modern counterparts.^29–33^

In its most basic form, microsimulation is a method for generating micro-level data, typically by combining individual- and aggregate-level datasets (i.e. population synthesis)^34^; this provides an estimated cross-sectional snapshot of a population. This synthetic population may then be statistically analysed to examine associations between its variables (as in ‘traditional’ data analysis) or, perhaps more interestingly, it can provide the foundation for a dynamic simulation model (either MSM or ABM). Both dynamic MSMs and ABMs simulate the evolution of heterogeneous individuals through time and potentially space. Each individual possesses a set of attributes (e.g. physical, socio-demographic, geographic), which may be updated at discrete time steps; in microsimulation models, in particular, individuals are often defined as belonging to one of a finite number of mutually exclusive and collectively exhaustive states (e.g. healthy, sick and dead), and events of interest are modelled as transitions from one state to another that occur according to a set of deterministic and/or stochastic rules (‘transition probabilities’) defined by the modeller.^35–37^ Conceptualized in this way, one may see parallels between the data-generating process represented by a DAG and the process by which individuals (and their attributes) evolve within the simulations.

Where MSMs and ABMs usually diverge, however, is in the level of complexity in the assumptions each adopts and adheres to regarding the underlying data-generating processes. A defining feature of ABMs—and what often separates them from MSMs—is the presence of interactions amongst individuals^34^; however, the distinction is primarily philosophical rather than methodological. Individuals within an ABM are explicitly conceptualized as *agents*—i.e. as autonomous, adaptive individuals with bounded rationality.^3^ Often this agency manifests in the form of responding to and making decisions influenced by other individuals within the simulation; such agent-to-agent interaction may give rise to what is referred to in the epidemiological literature as ‘interference’, and makes both representation of the scenario as a DAG and subsequent statistical estimation of causal effects considerably more complex^38–40^ because the focus is no longer limited to central locations (i.e. means) but rather the entire distribution of values for each variable as dictated by individual-level interactions. Within a standard DAG (e.g. Figure 1), each variable has a distribution of values across individuals that is determined by the variables that causally precede it; within an ABM, that distribution has an additional within-variable dependency on individual-level relationships. Thus, the data-generating process of an MSM is more easily represented by a DAG (as in Murray *et al*.^18^) than that of an ABM.

The potential of both MSMs and ABMs to evaluate counterfactual scenarios (or ‘what if’ scenarios^17^) should be immediately apparent. The modeller may alter e.g. one or more transition probabilities (or features of the agent-to-agent interaction, if applicable), cluster effects or somehow fix or limit the values allowable for any attribute, and then allow the effects of such perturbations to play out in the simulation. MSMs and ABMs inherently provide exchangeable units of analysis, as each simulated run serves as a counterfactual scenario given that the initial population remains unchanged.^16^

## How historical differences have informed philosophical and methodological differences

Examining the history of each method, as we have done in the previous section, is useful because historical knowledge is integral to understanding why each evolved in the particular discipline(s) it did and thus what types of causal questions it is well suited to addressing. After all, methods are simply tools developed to accomplish some particular objective; it is no coincidence that the three methods considered here have largely evolved in their separate disciplines. Hernan^41^ has provided a particularly interesting commentary on DAG-based regression modelling and simulation modelling, framing their differences in terms of their relative reliance on data vs theory—with DAG-informed models being more reliant on data and ABMs more on theory—and thus reflecting the relative value placed on data and theory within the disciplines in which they are typically used. We illuminate additional differences between the three methods that arise from their separate historical evolutions, including their relative focus on fixed vs random effects, and the timescales and timeframes in which they operate.

### Research questions

Due to its historical methodological foundations in the field of medicine, epidemiology (though arguably a social science) has tended to direct greater focus towards causal questions that lend themselves to experimentation, in an attempt to make inferences as independent as possible of theoretical arguments.^41^ Even when experimentation is infeasible, large quantities of (observational) individual-level data are collected and statistical methods (e.g. regression modelling) are employed with the aim of mathematically controlling for those factors that would typically be controlled via experimental manipulation. The recent revolution in graphical model theory has provided a theoretical foundation for causal data analysis that has historically been lacking, but it nevertheless remains that epidemiology is a data-loving science. Consequently, as noted by Hernan,^41^ minimizing (albeit not eliminating) the role of theory has necessitated addressing narrower causal questions. This is the context in which DAGs have been employed and in which the majority of methodological work is ongoing.^42^^,^^43^ Disciplines such as sociology and psychology, however, tend to be interested in answering broader, more theory-driven questions relating to phenomena for which data do not exist or may be difficult to measure or quantify (e.g. social norms); the theory-driven, data-generative nature of ABMs makes them more suitable for modelling such contexts. Economics—the primary realm of MSMs—falls somewhere in between, and indeed the discipline has shown a greater willingness to embrace graphical model-based methods (e.g. instrumental variable analysis^44^) than some of the ‘softer’ social sciences.

As an illustration of how use of the three methods differs, we consider obesity as a case study. The obesity epidemic has previously been characterized as containing many features of a complex system^2^^,^^3^^,^^45^ as well as elements from a wide variety of disciplines (e.g. biology, social policy, economics, psychology, geography, etc.); thus, it offers an ideal context for comparing the methods of interest. Box 2 provides a sample of the stated research objectives for published studies that have examined obesity using DAG-informed regression modelling, microsimulation modelling or agent-based modelling. Examination of Box 2 reveals several interesting distinctions between the methods; it also illustrates the observation by Hernan^41^ that DAG-informed regression modelling and agent-based modelling exist along a spectrum according to the relative weights given to data and theory, with microsimulation modelling providing a bridge between them.

**Box 2.** A sample of the stated research objectives for published studies that have examined obesity using DAG-informed regression modelling (* denotes use of a ‘g-method’^46^), microsimulation modelling or agent-based modelling

**Table:** 

DAG-informed regression modelling	Microsimulation modelling	Agent-based modelling
‘… to estimate the joint effects of obesity and smoking on all-cause mortality and investigate whether there were additive or multiplicative interactions.’^47^*	‘… to establish whether 52-week referral to an open-group weight-management programme would achieve greater weight loss and improvements in a range of health outcomes and be more cost-effective than the current practice of 12-week referrals.’^48^	‘To explore the role that economic segregation can have in creating income differences in healthy eating and to explore policy levers that may be appropriate for countering income disparities in diet.’^49^
‘… to estimate the independent causal effects of body mass index […] and physical activity on current asthma.’^50^*	‘…to estimate the expected impact of the [1-peso-per-litre] tax [on sugar-sweetened beverages] on body weight and on the prevalence of overweight, obesity and diabetes in Mexico.’^51^	‘… [to compare] the effects of targeting antiobesity interventions at the most connected individuals in a network with those targeting individuals at random.’^52^
‘… to study whether weight-related anthropometrics, changes in BMI SDS [standard deviation score] and physical activity at different ages in childhood are associated with atopic disease by late childhood.’^53^	‘… to estimate changes in calorie intake and physical activity necessary to achieve the Healthy People 2020 objective of reducing adult obesity prevalence from 33.9% to 30.5%.’^54^	‘… [to] simulate how a mass media and nutrition education campaign strengthening positive social norms about food consumption may potentially increase the proportion of the population who consume two or more servings of fruits and vegetables per day in NYC.’^55^
‘… to estimate the 26-year risk of CHD [coronary heart disease] under several hypothetical weight loss strategies.’^56^*	‘To assess the cost-utility of gastric bypass versus usual care for patients with severe obesity in Spain.’^57^	‘… [to explore] the efficacy of a policy that improved the quality of neighborhood schools in reducing racial disparities in obesity-related behaviour and the dependence of this effect on social network influence and norms.’^58^
‘… [to evaluate] the associations between early-life POP [persistent organic pollutant] exposures and body mass index.’^59^	‘To analyse the cost-effectiveness of bariatric surgery in severely obese (BMI ≥ 35 kg/m^2^) adults who have diabetes.’^60^	‘… to examine: a) the effects of social norms on school children’s BMI growth and fruit and vegetable (FV) consumption, and b) the effects of misperceptions of social norms on US children’s BMI growth.’^61^
‘… to assess the mediating role of anthropometric parameters in the relation of education and inflammation in the elderly.’^62^	‘To estimate the impact of three federal policies on childhood obesity prevalence in 2032, after 20 years of implementation.’^63^	‘… to examine the effects of different policies on unhealthy eating behaviors.’^64^
‘… to examine differences in the contribution of obesity measures to adenoma risk by race.’^65^	‘To determine the cost-effectiveness of gastric band surgery in overweight but not obese people who receive standard diabetes care.’^66^

The research questions addressed by DAG-informed regression modelling in Box 2 tend to be framed in terms of estimating the effect of a specific factor on a specific outcome. The concept of intervention is often implicit in these analyses (e.g. ‘If we were to intervene to alter exposure to early-life persistent organic pollutions, how would this affect BMI?’, as in Karlsen *et al*.^59^), but may also be explicit, as in Danaei *et al*.^56^ In fact, the example of Danaei *et al*.^56^ is particularly enlightening due to its specific use of the g-formula, which—as has previously been noted by Murray *et al*.^18^—is broadly equivalent to microsimulation, because it effectively simulates the joint distribution of the variables in a DAG that would have been observed had an intervention been enacted in which all individuals were exposed to the putative causal factor of interest.^46^

Researchers using microsimulation modelling tend to exclusively focus on estimating the effect of a specific *policy or intervention* on a target outcome and, often, determining its cost-effectiveness.^37^^,^^67^ Inherent in and integral to these analyses are specific comparisons between alternative intervention programmes. Given its history in the field of economics, it is perhaps unsurprising but nevertheless illustrative that microsimulation modelling is used for such analyses, particularly when contrasted with analyses using agent-based modelling.

The explicit evaluation of interventions in microsimulation modelling crosses over to agent-based modelling, with several of the stated research objectives in the third column of Box 2 referring to specific hypothetical policy interventions. However, unique to agent-based modelling analyses is their exploration of social phenomena (e.g. economic segregation, social norms) in the simulation framework. Thus, although they share considerable overlap methodologically, microsimulation and agent-based modelling are distinct in their underlying purposes and practical utility. Moreover, because agent-to-agent interactions give rise to greater complexity, ABMs often result in highly nonlinear and chaotic states and produce ‘emergent’ properties^68^; consequently, ABMs are less suited than MSMs to producing the detailed predictions often required by economists and policymakers, but arguably more suited to modelling naturally complex social phenomena.

### Fixed vs random effects

Another—perhaps underappreciated—distinction between DAG-informed regression models and ABMs is their relative focus on fixed vs random effects, which also arises from their distinct historical evolutions. A natural consequence of using DAG-informed regression models is that intense focus is directed towards modelling mean structures and estimating mean (fixed) effects as opposed to evaluating distributional properties and understanding complexity by examining variation and the patterns of natural heterogeneity. Although DAGs describe causal processes that could potentially manifest in infinitely many (parametric) ways, the use of regression models to interrogate causal questions and identify average causal effects makes focus on the distributional properties of the variables of interest effectively redundant. Moreover, their mathematical foundation is built on the assumption of no interference or spillover effects, and so the complexity and heterogeneity that define a complex system are often strictly controlled via study design or averaged out and largely overlooked (thereby treated more as a nuisance and mere ‘noise’ than of substantive interest in its own right). However, it is undeniable that there are myriad determinants of health and disease—particularly social ones^15^—that operate on many levels and in a complex fashion, and about which the ‘random’ structures (possibly arising from individual interactions) are of equal, if not greater, importance to the ‘fixed’ structures. Such determinants may be of great interest to epidemiologists, yet statistical modelling is limited in the insights it can provide into the potential complexity of random structures that contain spillover effects and interference. For these reasons, causal questions involving such complexities have tended to be relegated to the social sciences, in which greater emphasis is placed on theory as opposed to data (i.e. the realm of ABMs).

Foundationally, ABMs are theoretically very different from their statistical counterparts; as recognized by Oakes,^69^ the outcome of interest is primarily the process by which group phenomena emerge. From the (micro-)simulated processes of ABMs, patterns and properties of the system emerge; mean effects may be eventually derived, but the primary focus is on conceptualizing and modelling the system as a whole, and how individual *agency* and *heterogeneity* interact to give rise to aggregate patterns. Although ABMs have seen some use within epidemiology, this is largely confined to the study of infectious diseases^70–73^ in which there exist clear transmission mechanisms via individual interaction^74^ and in which it is widely recognized that the effects of interaction are a fundamental part of the causal mechanism and thus cannot be overlooked.^38^ Although the random effects arising from agent-to-agent interactions in ABMs are absent in MSMs, individuals remain the central focus of MSMs rather than average patterns. This individual-level focus allows the analysis of heterogeneity and distributional properties that might be masked by approaches considering only mean effects.^33^^,^^37^

### Timescale and timeframe

There also exists a large divergence between DAG-informed regression modelling and microsimulation/agent-based modelling with regard to how time is incorporated into the analyses—in terms of both scale and frame. Time is an inherent factor in any causal analysis, though there are infinitely many possibilities regarding the scale at which it is conceptualized and modelled. Because all models are abstractions of reality, both the salient features of a system and the frequency at which they are measured and represented are subjective choices that depend on context (and convenience, in the case of data-dependent analyses). For example, individual activity levels might be modelled every few seconds (as recorded by an activity monitor) to discover how exercise relates to heart rate during high-intensity interval training. However, such granularity would likely be unnecessary for determining how exercise relates to adipose tissue levels, in which case individual activity levels might be recorded as an average daily, weekly or monthly value; on the other hand, insufficient granularity of timescale (e.g. yearly or bi-yearly averages, or a one-time cross-sectional measurement) could have a detrimental impact on any analyses, as the circular feedback loop that occurs—typically on a much smaller timescale—between physical activity and obesity would be masked.

In general, the timescales upon which both methods operate are strikingly different. MSMs and ABMs tend to model much smaller timescales (e.g. days, weeks, months) than do statistical models because these are closer to the timescales upon which human behaviour and interactions generally operate, and upon which the effects of policy interventions might be realized. For ABMs in particular, in which agent-to-agent interactions are integral to the causal processes operating (e.g. for infectious diseases), modelling geolocation with high frequency is essential. Greater granularity of timescale enables the accumulation of emergent properties—although modelled in discrete time steps—to be approximately smoothed. Moreover, abstraction to larger scales has the potential to miss out on the complexity that these models seek to explore and/or explain and, because they are not as limited by data availability, they are able to explore phenomena in such granularity when the context requires it. Although DAG-based regression models are theoretically able to model such small timescales, their reliance on data (which tend to be collected infrequently, as in observational health studies, for instance) limits this in practice; they tend to be parameterized in a less granular fashion, which additionally serves their focus on mean effects and model parsimony.

Additionally, the timeframes in which the different models operate diverge. Because they are reliant upon data, DAG-based regression models exclusively model past events; the counterfactuals created are thus thought experiments about what *would have happened* had some condition been different. However, public health and epidemiological researchers are generally interested in estimating causal effects because they wish to intervene to alter (ideally beneficially) future health states; they may extrapolate the results of their statistical models to infer that what *would have happened* in the past is equivalent to what *would* happen in the future, but they do not explicitly model this. In contrast, MSMs and ABMs may be used to model both past and future events by utilizing and synthesizing historical data and estimates to make decisions about hypothetical future interventions; indeed, estimating the future impact of potential policy interventions has historically been fundamental to the utility of these methods.^33^^,^^37^^,^^75^

## Discussion

The identifying features of each of DAG-informed regression modelling, microsimulation modelling and agent-based modelling are briefly summarized in Table 1; we also include concise summaries of their accepted strengths and weakness.

**Table 1.** Brief summaries of the key features, strengths and weakness of each of DAG-informed regression modelling, microsimulation modelling and agent-based modelling. Note that the lists of strengths and weaknesses is not intended to be exhaustive

	DAG-informed regression modelling	Microsimulation modelling	Agent-based modelling
Short description/key features	Variables connected by causal pathways representing the data-generating process; used to inform statistical (regression) models	Simulated individuals that evolve over time, often transitioning between ‘states’	Simulated individuals that evolve over time and interact with one another, producing ‘emergent’ properties
Other common names/examples	G-methods^76^ (parametric g-formula, inverse probability of treatment weighting of marginal structural models, g-estimation of structural nested models)	Individual-based (simulation) models First-order Monte Carlo models^77^ State transition models^37^	Individual-based (simulation) models Dynamic (transmission) models^78^
Strengths	Backed by formal mathematics of graphical model theory Provide robust estimates of causal effects for clearly defined exposures and outcomes Assumptions underlying each model are transparent	Can evaluate the (future) effects of alternate intervention strategies Can combine parameter estimates from multiple datasets Greater focus on outcome distributions	Capable of modelling feedback loops and spillover effects Can incorporate hard-to-measure concepts and individual agency Capable of modelling future timeframes Greater focus on outcome distributions
Weaknesses	Require large individual-level datasets Not naturally suited to modelling longitudinal scenarios Primarily focus on mean (average) effects	Combination of parameter estimates from different populations may result in bias^18^ Small parameterization errors may be perpetuated throughout the simulation and result in large biases	Model complexity makes parameterization, calibration and validation difficult Lack of consensus about fundamental assumptions or under what circumstances causal effect estimates are valid^16^

As have previously been detailed, there exist substantive historical, theoretical and methodological differences between DAG-informed regression modelling, microsimulation modelling and agent-based modelling that make them suited to addressing different types of causal questions. DAG-informed regression modelling is appropriate for analyses in which the query of interest can be explicated in the traditional language of ‘exposures’ and ‘outcomes’ (e.g. ‘What is the effect of gastric bypass surgery [the exposure] on risk of diabetes [the outcome]?’), for which sufficient individual-level data are available on a suitable timescale for the causal processes of interest, and for which spillover effects and interference are thought to be negligible. Moreover, in terms of their practical utility in policy-making decisions, they are better suited to evaluating exposures/interventions whose effects can be safely assumed to be more or less transportable across time, so that the effects estimated from past data may be carried forward to the hypothetical future. When such conditions are met, DAG-informed approaches provide a robust method for causal inference whilst requiring relatively few assumptions, and offer a transparent means for communicating those assumptions.

At the other end of the spectrum, ABMs provide a means for modelling greater complexity—e.g. in the form of individual interactions and spillover effects—though they do so by requiring a greater number of assumptions. Moreover, because they model scenarios in which key variables of interest may not lend themselves to numerical representation, or in which observed data are not sufficiently granular in timescale to fully inform parameterization and/or enable effective validation, ABMs inherently contain greater uncertainty about the validity of their causal effect estimates.^77^^,^^79^^,^^80^ Here, MSMs offer a useful halfway house: they may be able to utilize the robust foundations of graphical causal models and also explore the effects of potentially complex interventions that occur over prolonged periods of time, possibly in the future. The results of Murray *et al*.^18^^,^^81^ (which demonstrate equivalence between the g-formula and microsimulation, and use the g-formula to inform microsimulation model parameters) represent the first endeavours to bring the mathematical robustness of graphical model theory to bear on simulation approaches. Further methodological research in this area promises to be fruitful.

## Funding

This work was supported by the Economic and Social Research Council (ES/J500215/1 to K.F.A.) and the Higher Education Funding Council for England.

**Conflict of interest:** None declared.
